# Response to Selective RET Inhibition With LOXO-292 in a Patient With *RET* Fusion-Positive Lung Cancer With Leptomeningeal Metastases

**DOI:** 10.1200/PO.19.00021

**Published:** 2019-06-03

**Authors:** Robin Guo, Mark Schreyer, Jason C. Chang, S. Michael Rothenberg, Dahlia Henry, Paolo Cotzia, Mark G. Kris, Natasha Rekhtman, Robert J. Young, David M. Hyman, Alexander Drilon

**Affiliations:** ^1^ Memorial Sloan Kettering Cancer Center, New York, NY; ^2^ Loxo Oncology, Stamford, CT; ^3^ New York University Langone Medical Center, New York, NY; ^4^ Weill Cornell Medical College, New York, NY

## INTRODUCTION

The development of leptomeningeal metastases is a poor prognostic factor in patients with advanced cancers.^1-3^ In non–small-cell lung cancers (NSCLCs), median overall survival of patients from the diagnosis of leptomeningeal disease is 1 to 2 months without treatment and up to 8 months with systemic therapy.^4-6^ Furthermore, patients with leptomeningeal disease have historically had limited access to novel therapies in clinical trials. Recent efforts from many groups, including the European Society for Medical Oncology and the US Food and Drug Administration (FDA), have encouraged the inclusion of patients with leptomeningeal metastases in clinical trials, in addition to promoting standardization of intracranial response assessments.^7-9^ These efforts are crucial given that many investigational agents have substantial CNS activity and may improve outcomes in driver-positive cancers with leptomeningeal involvement.^5,10^

*RET* fusions are actionable oncogenic drivers that are identified in 1% to 2% of NSCLCs.^11,12^ To date, chemotherapy and/or immunotherapy remain the only approved systemic therapies for these cancers. Multikinase inhibitors with activity against RET (eg, cabozantinib or vandetanib) were repurposed to treat patients with *RET* fusion-positive lung cancers. Although these agents were found to be active in a subset of these patients, outcomes are modest compared with targeted therapies in other driver-positive lung cancers, and intracranial activity is poor.^13,14^ Selective RET inhibitors currently in development, such as LOXO-292 and BLU-667, have improved outcomes for patients with *RET* fusion-positive cancers because of increased potency and less off-target toxicity.^15,16^ In September of 2018, LOXO-292 received Breakthrough Therapy designation from the FDA for treatment of patients with metastatic *RET* fusion-positive NSCLCs (as well as *RET* fusion-positive thyroid cancers and *RET*-mutant medullary thyroid cancer). In addition, confirmed intracranial responses and durable disease control have been achieved in patients with brain metastases in an ongoing phase I/II trial of LOXO-292 for patients with *RET* fusion-positive cancers.^15^ Its activity in leptomeningeal disease, however, has not previously been characterized. In this article, we describe a patient with a *RET* fusion-positive lung cancer with brain and leptomeningeal metastases who had an impactful intracranial response to selective RET inhibition with LOXO-292.

## CASE REPORT

**FIG 1.**
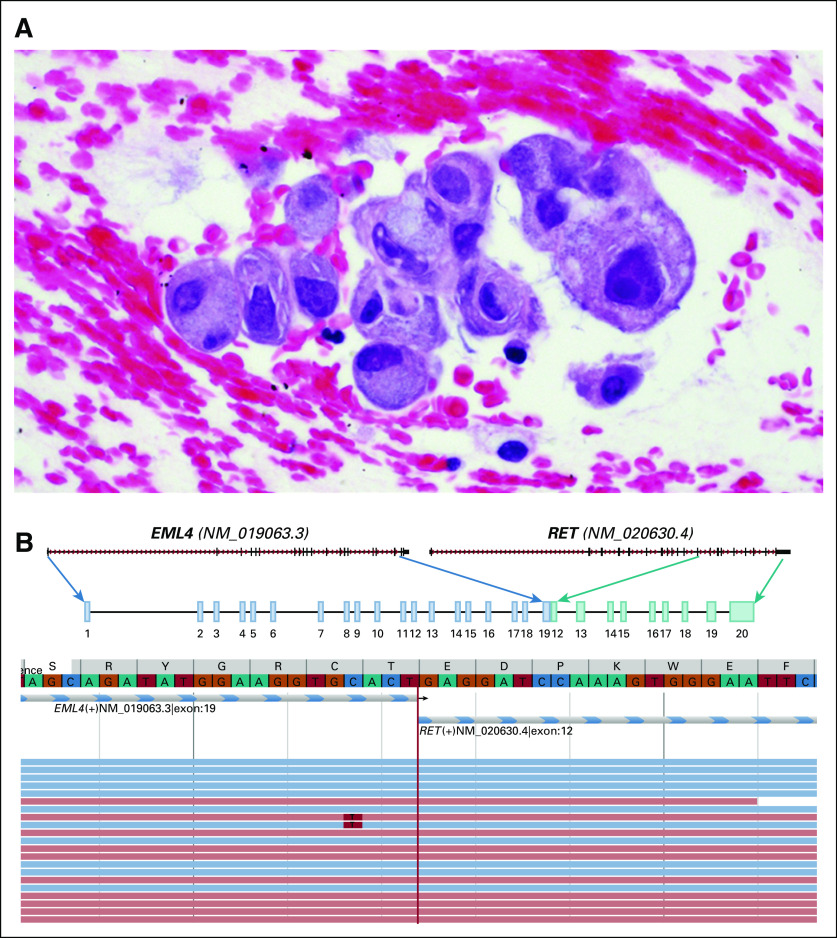
Histologic and molecular features of a *RET* fusion-positive lung cancer. (A) A hematoxylin and eosin–stained section from a cell block of a fine-needle aspiration specimen from a lower paratracheal lymph node confirmed a diagnosis of lung adenocarcinoma. Clusters of malignant epithelial cells with signet-ring cell morphology (eccentrically placed nuclei, focally prominent nucleoli, and abundant amount of cytoplasm containing grayish-blue mucin) are shown. (B) An in-frame *RET* fusion containing the RET tyrosine kinase domain was identified in extracted DNA from this sample by broad, hybrid capture–based next-generation sequencing using the Memorial Sloan Kettering Integrated Mutation Profiling of Actionable Cancer Targets—MSK-IMPACT— and Illumina HiSeq 2500 (Illumina, San Diego, CA). Exon 19 of the 5′ upstream gene partner *EML4* was fused to exon 12 of 3′ *RET*. This *EML4-RET* fusion was confirmed using an RNA-based anchored multiplex polymerase chain reaction (ARCHER, Illumina MiSeq [ARCHER, Boulder, CO]).

A 33-year-old female never-smoker presented with cough and dyspnea. Computed and positron-emission tomography imaging revealed a hypermetabolic 4.8-cm right lower lobe mass, mediastinal and hilar adenopathy, and osseous metastases involving L1, the sacrum, and the left anterolateral sixth rib. Magnetic resonance imaging (MRI) of the brain showed three subcentimeter enhancing foci in the right precentral gyrus, right parietal lobe, and left temporal lobe. Endobronchial biopsy of an R4 lymph node revealed adenocarcinoma with signet ring cell features (Fig 1A). Tumor cells were positive for TTF-1 and negative for p40 by immunohistochemistry. Broad, hybrid capture–based next-generation sequencing using the Memorial Sloan Kettering Integrated Mutation Profiling of Actionable Cancer Targets—MSK-IMPACT—and Illumina HiSeq 2500 (Illumina, San Diego, CA)^17^ identified an *EML4-RET* fusion (Fig 1B) in addition to a *TP53* p.P142Tfs*5 frameshift mutation. This *EML4*-*RET* fusion was confirmed using a targeted RNA-based anchored multiplex polymerase chain reaction—ARCHER Fusion Assay (ARCHER, Boulder, CO).

With identification of the *RET* fusion, the patient was treated with the investigational anti-RET multikinase inhibitor RXDX-105.^18,19^ Although a confirmed partial response was initially achieved (a near-complete response in her brain metastases), her course was marked by isolated asymptomatic intracranial progression requiring multiple radiation treatments. A year after initiating therapy, she underwent stereotactic radiosurgery (21 Gy) to five new enhancing subcentimeter parenchymal metastases. Seven months later, she developed further intracranial progression requiring 42 Gy of stereotactic radiosurgery to seven additional lesions. Given absence of extracranial disease progression, RXDX-105 was continued.

**FIG 2.**
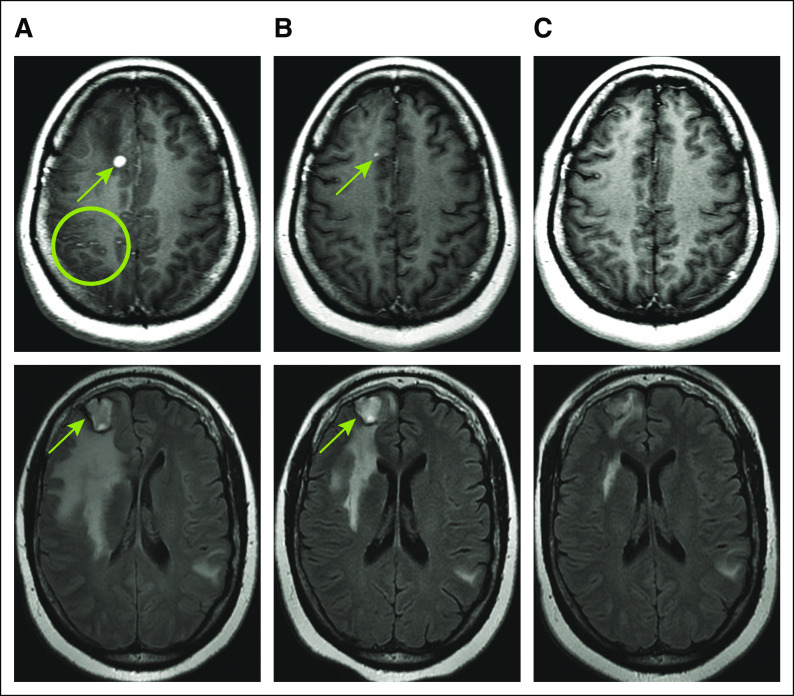
Intracranial response to LOXO-292. Magnetic resonance imaging axial brain images are shown (A) at baseline, (B) 5 weeks, and (C) 21 weeks after the initiation of LOXO-292 therapy in a patient with an *EML4-RET* fusion-positive lung cancer. (A) Scattered leptomeningeal enhancement (green circle, lower left), consistent with untreated leptomeningeal metastases are noted. (B and C) The patient’s leptomeningeal disease completely resolved radiologically with LOXO-292 therapy. A representative right superior medial and right lower frontal intraparenchymal metastasis (green arrows) regressed with LOXO-292 therapy, along with several other metastases followed on serial imaging. A confirmed partial intracranial response by RECIST (Response Evaluation Criteria in Solid Tumors) v1.1 and a complete response in leptomeningeal disease by Response Assessment in Neuro-Oncology were achieved. The patient continues to receive LOXO-292 with ongoing disease control at 10.8 months.

**FIG 3.**
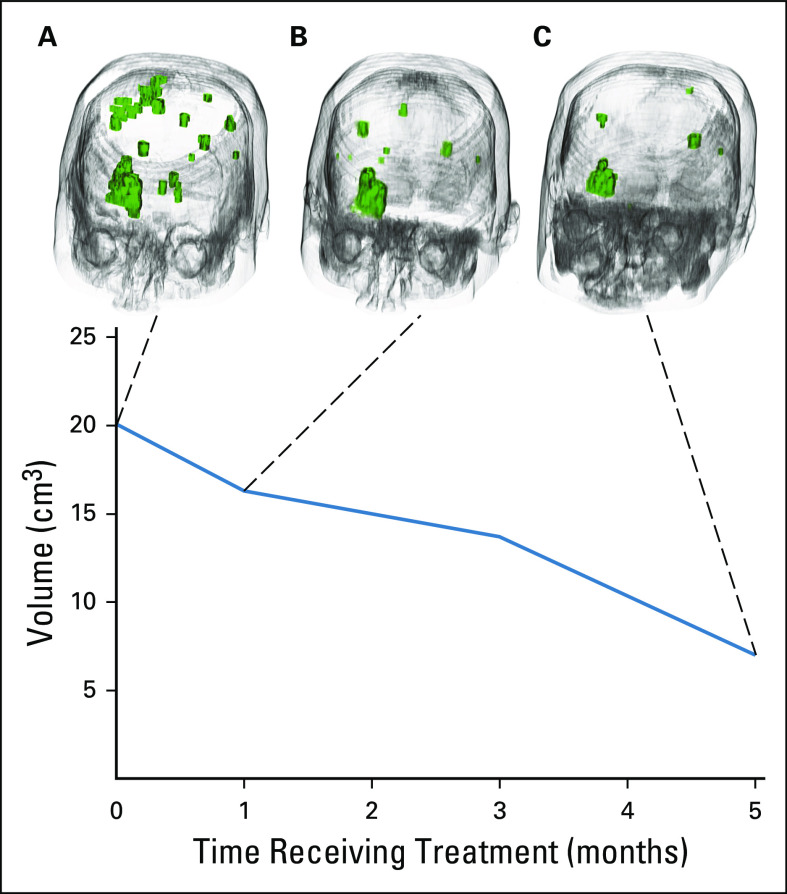
Volumetric response assessments. Volumetric three-dimensional magnetic resonance imaging analyses were performed on serial imaging performed at (A) baseline, (B) 5 weeks, and (C) 21 weeks. In the upper panels, both leptomeningeal and parenchymal metastases are visualized as green three-dimensional figures. LOXO-292 therapy resulted in a substantial decrease in volumetric disease over time. Maximal volumetric disease regression of 65% was achieved at 21 weeks in the graph of total volume over time on LOXO-292 therapy as shown in the bottom panel. Both leptomeningeal disease and brain metastases are included in the baseline volume calculation. Leptomeningeal enhancement could not be detected on subsequent scans.

Four months later, the patient developed symptomatic progression of brain metastases and new leptomeningeal disease. She presented with left facial, tongue, and upper extremity tingling and worsening neck pain. These symptoms were deemed to be secondary to leptomeningeal disease that was identified radiologically in the right hemisphere, predominantly in the right parietal lobe (Fig 2A; top panel), recognizing that nonradiologically apparent disease was likely present in other areas.^8^ Multiple brain metastases had also increased (largest measuring 2.7 cm in the right frontal lobe; Fig 2A; bottom panel). Using volumetric three-dimensional MRI, the total volume of radiologically significant intracranial metastases was 20.1 cm^3^ (Fig 3A). A lumbar puncture was recommended, but the patient declined; a brain biopsy to potentially determine the mechanism of resistance to RXDX-105 was not deemed safe. Extracranial imaging again showed no evidence of disease.

Given that the patient was highly symptomatic with progressive symptoms, a single-patient use protocol of LOXO-292 was approved by the FDA and institutional review board. The patient provided written informed consent before enrollment, and LOXO-292 was administered orally at 160 mg twice daily. This dose was selected based on preliminary safety and efficacy results from an ongoing phase I/II trial of the drug (ClinicalTrials.gov identifier: NCT03157128). Imaging assessments (MRI of the brain and computed tomography of the chest, abdomen, and pelvis) were performed every 8 weeks. Response was assessed by RECIST (Response Evaluation Criteria in Solid Tumors) version 1.1.^20^ Response of leptomeningeal metastases was assessed in accordance with Response Assessment in Neuro-Oncology criteria.^8^ Additional volumetric three-dimensional imaging was performed on subsequent scans (Sloan Kettering Advanced Imaging Lab, SAIL; Figs 3B and 3C).

A clinical response to therapy was achieved within the first week of therapy, with improvement and subsequent resolution of the patient’s neurologic symptoms. This was accompanied by a confirmed radiologic response to therapy. A partial response in the brain by RECIST v1.1 was achieved at follow-up imaging assessment at 16 weeks and confirmed by subsequent imaging. In addition, LOXO-292 therapy achieved complete resolution of leptomeningeal enhancement, with a Response Assessment in Neuro-Oncology leptomeningeal score dropping from 1 at baseline to 0 at 8 weeks. Volumetric assessment revealed a continued decrease in the total volume of significant intracranial disease (leptomeningeal and parenchymal), with a maximal shrinkage of 65% (from 20.1 cm^3^ at baseline to 7 cm^3^) at 5 months (Fig 3C).

The patient continues to receive therapy with LOXO-292 at 10.8 months, with ongoing radiologic disease control and no neurologic symptoms. She reports only grade 1 fatigue. There continues to be no evidence of extracranial disease with imaging.

## DISCUSSION

The development of leptomeningeal disease can represent a devastating complication in patients across a wide variety of different cancers. Conventional chemotherapy or radiotherapy can be used in select cases, but outcomes are marginal to modest at best. In NSCLCs with leptomeningeal metastases, there is no consensus on the use of whole-brain radiotherapy, because it has not been shown to consistently improve survival.^1,3^ Although systemic chemotherapy with more contemporary regimens (including pemetrexed and/or bevacizumab) and intrathecal chemotherapy have been shown to improve outcomes in select series, the development of new agents with higher response rates and more durable disease control continues to represent an unmet need for many patients.^4,6^

This report represents the first description, to our knowledge, of leptomeningeal metastases responding to any systemic therapy in a patient with a *RET* fusion-positive cancer. A brisk and durable ongoing response to LOXO-292 was achieved in a patient with a *RET* fusion-positive lung cancer who had notable disease progression while receiving a prior multikinase inhibitor and multiple prior stereotactic radiosurgery treatments. These outcomes are consistent with the previously reported activity of LOXO-292 in parenchymal brain metastases. In preliminary data from an ongoing phase I/II trial (ClinicalTrials.gov identifier: NCT03157128), all four patients with untreated measurable parenchymal metastases had confirmed intracranial responses to therapy accompanied by overall disease control.^15^ Durable intracranial and extracranial disease control was likewise established in several other patients with untreated nonmeasurable brain metastases before LOXO-292 therapy.

The activity of LOXO-292 in the CNS can be attributed to several factors. The drug is active preclinically, with oral dosing in an orthotopic mouse model of a *RET* fusion-positive patient-derived tumor implanted into the brain.^21,22^ Its potency and selectivity for RET are also likely contributory. Using a highly active agent in the CNS is crucial in *RET* fusion-positive lung cancers, because close to 25% of patients present with intracranial disease at baseline, whereas the lifetime prevalence of brain metastases approaches 50%.^14^ In addition, LOXO-292 was designed to target potential resistance mechanisms that can emerge from prior multikinase inhibitor use, such as *RET* V804M/L gatekeeper substitutions. Although the profile of this patient’s resistance to RXDX-105 is unknown (it was not deemed safe to do repeat biopsies of her brain metastases, and the patient declined a lumbar puncture), LOXO-292 clearly re-established disease control after prior multikinase inhibitor therapy, consistent with results seen with other patients treated in the ongoing phase I/II trial.^21^

Taking these observations into context with data from other driver-positive lung cancers, select next-generation tyrosine kinase inhibitors (TKIs) with intracranial activity arguably represent the optimal agents to use, not only to treat preexisting intracranial disease but also to prevent its emergence.^23,24^ Osimertinib, a third-generation epidermal growth factor receptor (EGFR) TKI, demonstrated impressive intracranial activity in patients with *EGFR*-mutant lung cancers, not only in patients with parenchymal brain metastases but likewise in those with leptomeningeal disease.^25-27^ Similarly, later-generation, more potent anaplastic lymphoma kinase (ALK) TKIs, such as alectinib, ceritinib, brigatinib, or lorlatinib, resulted in higher intracranial response rates, durable intracranial disease control, and a delay in the development of progression in the CNS compared with crizotinib (for which the CNS is a common site of progression) in *ALK* fusion-positive lung cancers.^24,28,29-32^

Finally, the intracranial activity of the selective RET inhibitor LOXO-292 has substantial implications beyond NSCLCs. The drug is currently being explored and has been shown to be active across multiple RET-dependent tumors.^21^ *RET* fusions are also identified in papillary thyroid, anaplastic thyroid, colorectal, pancreatic, and breast cancers; Spitzoid neoplasms; and chronic myeloproliferative neoplasms.^11^ Somatic and germline-activating *RET* mutations are likewise actionable drivers of oncogenesis that are identified in medullary thyroid cancers and potentially other malignancies.^33^ Although the frequency at which intracranial metastases present is lower for many of these other cancers compared with NSCLCs, metastatic disease in the CNS can occur in some cases.^34^

In conclusion, selective RET inhibition with LOXO-292 achieved a clinically meaningful and confirmed response in a patient with a *RET* fusion-positive lung cancer with leptomeningeal disease and heavily pretreated brain metastases. Although additional confirmation of this activity will help elucidate overall intracranial disease outcomes, this report underscores the potential of selective RET inhibition as a means of treating and preventing the occurrence of disease in the CNS in patients with RET-dependent cancers of any histology.
